# An Unexpected Complication of Hip Arthroplasty: Knee Dislocation

**DOI:** 10.1155/2015/294187

**Published:** 2015-08-10

**Authors:** Serdar Yilmaz, Deniz Cankaya, Alper Deveci, Mahmut Ozdemir, Murat Bozkurt

**Affiliations:** ^1^Department of Orthopaedics and Traumatology, Ankara Numune Training and Research Hospital, 06100 Ankara, Turkey; ^2^Department of Orthopaedics and Traumatology, Ankara Kecioren Training and Research Hospital, 06280 Ankara, Turkey; ^3^Department of Orthopaedics and Traumatology, Ankara Ataturk Training and Research Hospital, 06800 Ankara, Turkey

## Abstract

An increasing number of patients with hip fracture have been seen with osteoporosis associated with osteoarthritis. Although knee dislocation is related to high-energy trauma, low-grade injuries can also lead to knee dislocation which is defined as “ultra-low velocity dislocation.” The case reported here is of an 82-year-old patient who presented with a left intertrochanteric hip fracture. Partial arthroplasty was planned because of osteoporosis. In the course of surgery, degenerative arthritic knee was dislocated during the hip reduction maneuver with the application of long traction. The neurovascular examination was intact, but the knee was grossly unstable and was dislocated even in a brace; thus a hinged knee prosthesis was applied nine days after surgery. The patient was mobilized with crutches after the knee prosthesis but exercise tolerance was diminished. In conclusion, it should be emphasized that overtraction must be avoided during the hip reduction maneuver in patients with advanced osteoarthritic knee.

## 1. Introduction

Hip fractures are a common problem of elderly people. The number of hip fractures is thought to have increased as the overall population ages [1–3]. Although the rationale of the treatment is to maintain the mobility of the patient, the morbidity and mortality rate after hip fracture is high [3, 4]. Complications such as hip dislocation, infection, periprosthetic fracture, deep vein thrombosis, and pulmonary embolism can be seen after surgery [5].

Hip arthroplasty in elderly patients is problematic because of osteoporosis. The expected stability is low and periprosthetic fractures can occur during the surgery [5, 6]. An increasing number of patients have been seen with osteoporosis associated with osteoarthritis in elderly patients with a hip fracture [6]. Of individuals older than 60 years, 10% of males and 18% of females are thought to have severe arthritis [6]. The degeneration of the ligaments in arthritic patients leads to mechanical weakness and this can be a problem during surgery [7–9]. The surgery must be accomplished under these circumstances in hip fracture patients.

The case reported here is of a patient with knee dislocation with underlying knee osteoarthritis which occurred during partial hip arthroplasty in the hip reduction maneuver after hip fracture. To the best of our knowledge, this is the only case of its type reported in the literature. The patient's family were informed that data from the case would be submitted for publication and gave their consent.

## 2. Case Report

**Figure 1:**
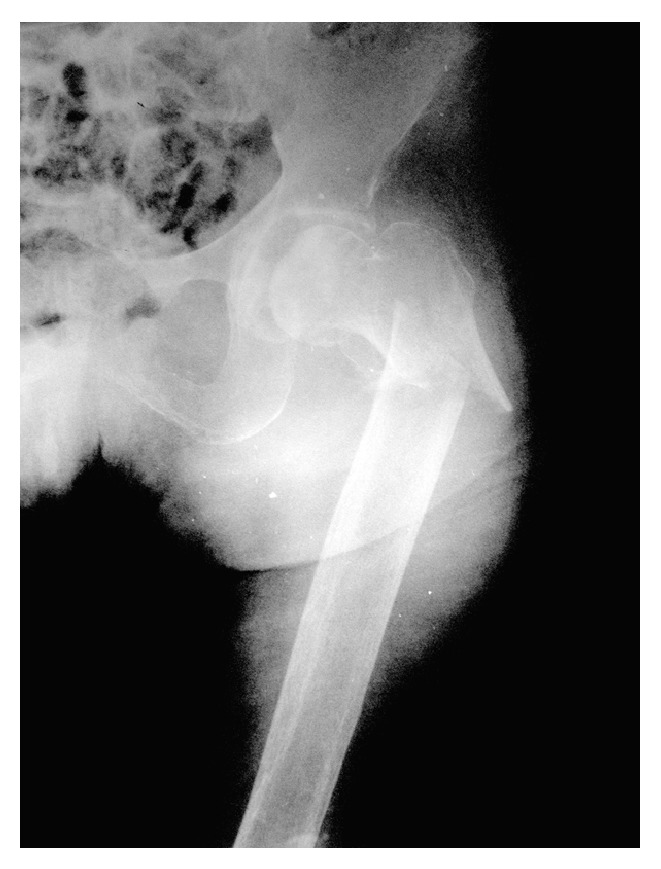
Left intertrochanteric hip fracture seen on radiography. It was understood from the widened femoral canal that osteoporosis was associated with the fracture.

**Figure 2:**
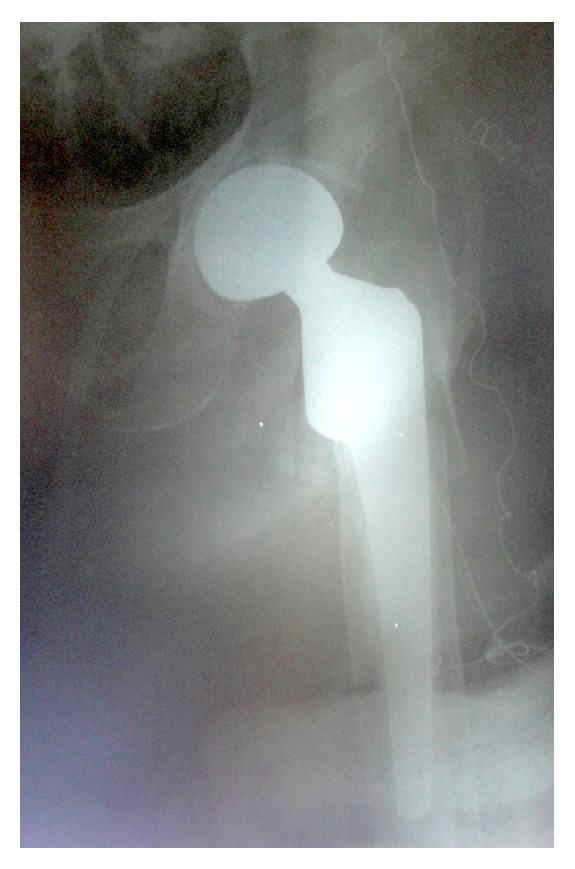
Partial cemented hip arthroplasty was applied with a calcar replacing femoral stem. The femoral stem position was satisfactory in relation to the hip center.

**Figure 3:**
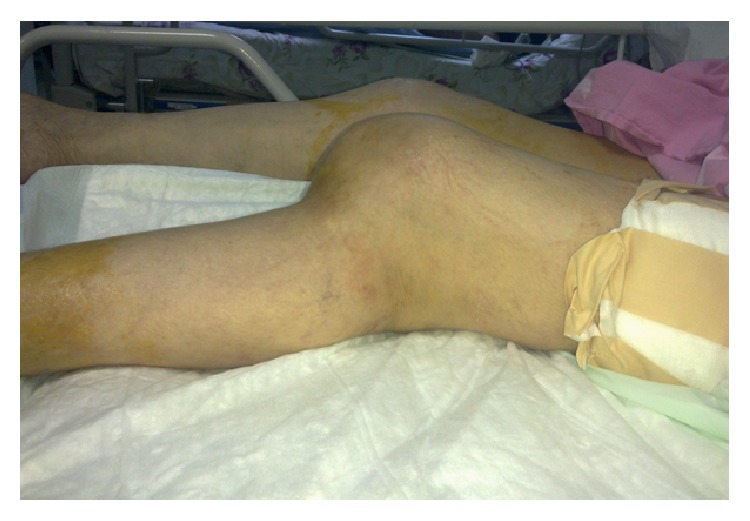
The deformed knee indicating posterior knee dislocation.

**Figure 4:**
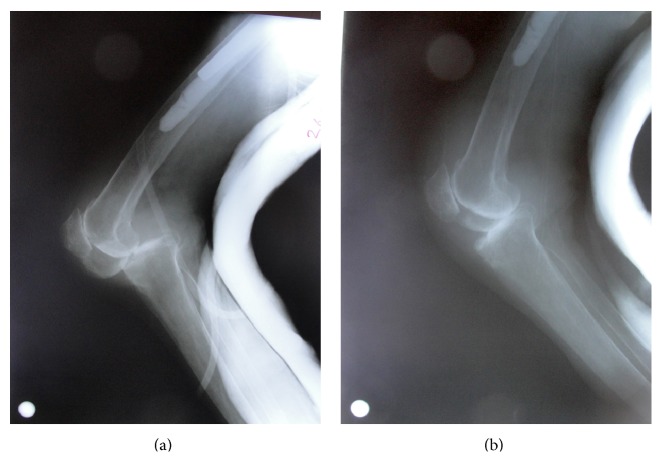
Posterior knee dislocation seen on radiography before and after reduction with underlying knee osteoarthritis.

**Figure 5:**
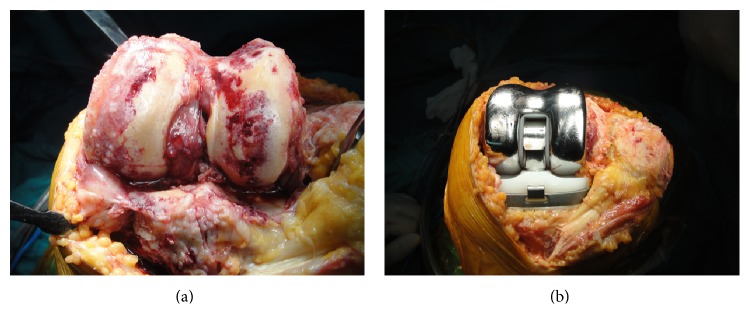
Degenerative arthritis and torn cruciate ligaments were seen and a rotating hinged knee prosthesis was applied.

**Figure 6:**
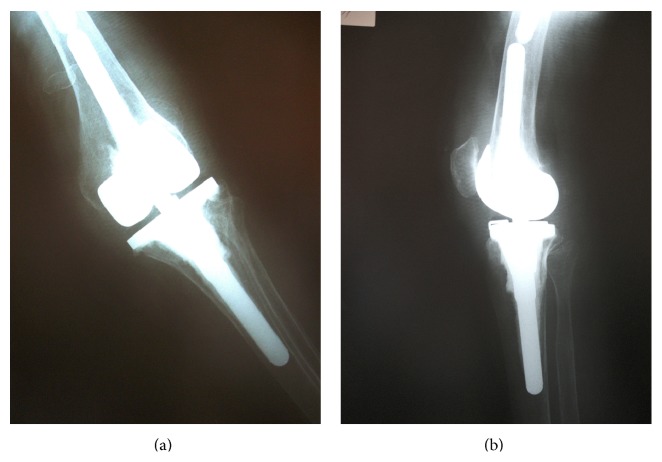
Radiography of the hinged knee prosthesis was satisfactory.

An 82-year-old female patient complaining of left hip pain after a simple fall was admitted to our hospital. There was pain and tenderness in the left groin and the patient could not stand on the left leg. The patient was mobilized with a cane before the trauma and body mass index was 23.8. The radiography revealed an intertrochanteric hip fracture (Figure 1) and the patient was hospitalized. After preoperative preparations, it was decided to perform hip arthroplasty instead of fixation because of severe osteoporosis and the low adaptive cooperation of this low-demand patient. Bipolar hip arthroplasty was applied with a posterolateral incision under spinal anesthesia. A cemented prosthesis was applied because of insufficient stability during the operation. After the femoral stem and the bipolar head were placed, an assistant applied traction to reduce the hip. The reduction was difficult to achieve and required aggressive reduction maneuvers. Hip radiography taken in the operating room was satisfactory and the femoral stem was not high placed (Figure 2). During the operation both legs were draped with elastic bandages and the knee was overlooked. After the operation when the patient was transferred to the bed, the left knee was seen to be deformed (Figure 3). Knee dislocation with underlying knee osteoarthritis was detected on radiography (Figure 4). The neurovascular evaluation was intact and the vascular continuity was confirmed with Doppler ultrasonography. However, the knee was grossly unstable so that it dislocated even in the cast or a knee brace. Treatment with a rotating hinged knee prosthesis was planned for the dislocated knee to be able to mobilize the patient as soon as possible. Surgery was applied 9 days after the first operation (Figure 5). A hinged knee prosthesis was applied and no complications were seen postoperatively (Figure 6). After the operation the patient was encouraged to comply with knee and hip strengthening and range of motion exercises. The patient was mobilized with a walker. It was subsequently learned from the family that the patient died due to cardiac arrest 7 months postoperatively.

## 3. Discussion

Hip fractures lead to substantial morbidity and mortality in geriatric patients [2–4]. Associated medical comorbidities also play a role in the survival of this population [4]. Degenerative arthritis is a common problem and worsens the condition of these elderly patients [5, 6]. Through degeneration of the ligaments and disturbance of the knee axis, the stability of the knee is diminished in patients with knee osteoarthritis [10, 11]. Although knee dislocation usually results from high-energy trauma, relatively low-grade trauma injuries can occur in arthritic patients [12].

Knee dislocation is a potentially devastating injury with a reported high rate of neurovascular injury [12]. This is usually related to high-energy trauma, but, in obese female patients, knee dislocation can occur, often from a simple fall, and this is defined as an “ultra-low velocity dislocation” [12]. This condition is associated with higher risks of neurovascular injuries and has more significant perioperative complications in obese patients. Although no relationship has been reported between degenerative arthritis and ultra-low velocity knee dislocation, some of these dislocations can be considered to be a consequence of the degenerative process of the knee.

It has been reported that degenerative and traumatic changes occur in the cruciate ligaments in osteoarthritis [9]. Cadaver studies evaluating the relationship of the PCL and degenerative osteoarthritis have reported few changes in the PCL with ageing if the knee was otherwise normal. However, the ACL and PCL showed serious degeneration in degenerative knees [7–9, 13]. The degeneration of the ligaments associated with degenerative arthritis leads to mechanical failure. This could be considered a factor in ultra-low velocity knee dislocation. The current case was not an obese patient unlike the general ultra-low velocity knee dislocation patients. In the only study in the literature about knee dislocation associated with knee osteoarthritis, Citak et al. reported a patient with osteoarthritis of the knee which progressively dislocated in a year spontaneously without any trauma [14].

There have been no previous reports in the literature of knee dislocation with underlying knee osteoarthritis occurring during hip arthroplasty in the course of the hip reduction maneuver and traction. The degeneration of the knee ligaments lowered the pull-out strength, so relatively small forces such as long traction can cause the dislocation of the knee [13]. Therefore, the femoral stem level must be carefully determined during the operation to avoid overtraction of the leg [15]. A high placed femoral stem requires vigorous traction to reduce the hip and may therefore cause knee dislocation in a patient with degenerative arthritis, as in the current case.

In conclusion, femoral stem position must be carefully determined in hip arthroplasty to avoid overtraction to the leg during the hip reduction maneuver especially in patients with knee osteoarthritis. Knee dislocation must be kept in mind during hip arthroplasty in cases of advanced knee arthritis.
